# Nasal Immunization Using Chitosan Nanoparticles with Glycoprotein B of Murine Cytomegalovirus

**DOI:** 10.4014/jmb.2308.08008

**Published:** 2023-12-22

**Authors:** Marcela Slovakova, Sylva Janovska, Radek Sleha, Vera Radochova, Alexandra Hatala, Nikola Mannova, Radovan Metelka, Ludovit Pudelka, Pavel Bostik

**Affiliations:** 1 University of Defence, Faculty of Military Health Sciences, Hradec Kralove 50001, Czech Republic; 2 University of Pardubice, Faculty of Chemical Technology, Pardubice 53201, Czech Republic; 3 Charles University, Faculty of Medicine in Hradec Kralove, Hradec Kralove 50001, Czech Republic

**Keywords:** Nanoparticles, vaccine, nasal administration, cytomegalovirus, murine

## Abstract

The use of nanoparticles as a delivery system for a specific antigen could solve many limitations of mucosal vaccine applications, such as low immunogenicity, or antigen protection and stabilization. In this study, we tested the ability of nasally administered chitosan nanoparticles loaded with glycoprotein B of murine cytomegalovirus to induce an immune response in an animal model. The choice of chitosan nanoparticle type was made by in vitro evaluation of sorption efficiency and antigen release. Three types of chitosan nanoparticles were prepared: crosslinked with tripolyphosphate, coated with hyaluronic acid, and in complex with polycaprolactone. The hydrodynamic size of the nanoparticles by dynamic light scattering, zeta potential, Fourier transform infrared spectroscopy, scanning electron microscopy, stability, loading efficiency, and release kinetics with ovalbumin were evaluated. Balb/c mice were immunized intranasally using the three-dose protocol with nanoparticles, gB, and adjuvants Poly(I:C) and CpG ODN. Subsequently, the humoral and cell-mediated antigen-specific immune response was determined. On the basis of the properties of the tested nanoparticles, the cross-linked nanoparticles were considered optimal for further investigation. The results show that nanoparticles with Poly(I:C) and with gB alone raised IgG antibody levels above the negative control. In the case of mucosal IgA, only gB alone weakly induced the production of IgA antibodies compared to saline-immunized mice. The number of activated cells increased slightly in mice immunized with nanoparticles and gB compared to those immunized with gB alone or to negative control. The results demonstrated that chitosan nanoparticles could have potential in the development of mucosal vaccines.

## Introduction

The chitosan nanoparticle platform has recently been intensively studied as an antigen delivery system for vaccines. Chitosan is a linear polysaccharide that is obtained by a programmable degree of deacetylation of natural chitin. It exhibits a number of positive characteristics, such as biocompatibility, biodegradability, and low toxicity [1]. Moreover, it possesses a promising ability to induce both humoral and cell-mediated immune responses and could be used as an immunological adjuvant [2]. Chitosan nanoparticles (ChiNPs) are easily formed by ionic cross-linking and spontaneous gelation in aqueous solutions on the basis of a reaction between chitosan amine groups and phosphate groups of tripolyphosphate (TPP) [3]. The crucial factors that affect the use of NPs as an antigen delivery system are their chemical and physical properties. The shape, size and surface charge are especially important parameters for an interaction with antigens or immune cells. Chitosan has a positive charge due to the presence of weakly basic amino groups of N-acetyl-D-glucosamine monomers [4]. This positivity is an advantage because ChiNPs can interact with negatively charged sites on cell surfaces [3]. Smaller particles (20-200 nm) can directly drain to lymph nodes, whereas larger particles (>200 nm) are taken up by macrophages or dendritic cells [5, 6].

Human cytomegalovirus (HCMV) is an enveloped virus belonging to the beta-herpesvirus family. It is distributed worldwide and is a significant cause of morbidity and mortality in newborns and immunocompromised adults [7]. HCMV infections in women during pregnancy can cause irreversible fetal neurodevelopmental abnormalities with severe consequences, such as mental retardation, cerebral palsy, and hearing loss. Although antiviral therapy is available and treatment with nucleoside analogs can improve the prognosis in immunocompromised patients with HCMV infections, the development of a CMV vaccine still remains the most promising strategy for preventing CMV infections. The main glycoprotein complexes on the virus surface represent important antigenic determinants that are utilized as antigenic targets for vaccine development in many clinical studies. The most potent of these is glycoprotein B (gB), which could provide immune protection against infection [7]. However, the other glycoproteins (gH, gM, gN, and gL) also represent important targets for virus neutralization. It has been demonstrated that natural HCMV infection induces a potent immune response comprising antibodies as well as CD4^+^ and CD8^+^ T cells [8]. Preexisting antibodies were observed to protect against CMV infection in monkeys, while a depletion of CD4^+^ cells could lead to an increase in viral transmission [9, 10]. Thus, for a vaccine to be effective it is necessary to induce a combination of both cellular and humoral immunity [11].

Due to the strict species specificity of CMV infection, there is no animal model available for studying HCMV infection and immunity. The most widely used is a murine model of mouse cytomegalovirus infection (MCMV). In this study we evaluated the ability of ChiNPs coated with envelope antigen of MCMV to induce the immune response after intranasal administration. The findings presented here provide new insights into the potential of ChiNPs as an antigen delivery system in a vaccine against cytomegalovirus.

## Materials and Methods

### Chemicals and Reagents

Low-molecular-weight chitosan (50–190 Da), sodium tripolyphosphate, polycaprolactone ~ 14,000 Da (PCL), isopropyl β-D-1-thiogalactopyranoside (IPTG), LB medium, albumin from chicken egg white (Ova), urea, Tris buffer and Tween 80 were obtained from Sigma-Aldrich (USA). Chitosan (> 95% deacetylate, high quality ChitoClear) was obtained from Primex (Iceland). Fluorescein isothiocyanate (FITC) MW 389,382 g/mol was obtained from Thermo Fisher (Czech Republic). Hyaluronic Acid HYSILK (Mw ~ 150–350 kDa) was purchased from Contipro (Dolni Dobrouc, Czech Republic). CpG ODN and Poly (I:C) were from InvivoGen (France).

### Recombinant Protein Synthesis

The expression of MCMV gB was performed in the ClearColi BL21 (DE3) strain. The inclusion bodies containing MCMV gB were washed and solubilized in 8 M urea buffer. Subsequently, the MCMV gB was purified by affinity chromatography using TALON metal affinity resins (Takara, France) according to the manufacturer’s instructions.

### Nanoparticle Preparation

ChiNPs were prepared by ionic gelation with TPP cross-linking as previously described [4], with the following modifications. Briefly, the prepared 0.2% solution in 0.025 M acetic buffer (pH 5.2) was filtered (0.45 μm). Subsequently, ChiNPs were spontaneously obtained when one volume part of the TPP solution (1 mg/ml) was added dropwise to the 2 volume parts of the chitosan solution under magnetic stirring (750 rpm) at room temperature. The mixture was stirred for an additional 30 min and then dialyzed in dialysis bags (Serva, Germany, MWCO 3,500 Da). Chitosan nanoparticles coated with hyaluronic acid (ChiNPs_HA) were prepared according to the modification of the protocol described previously [12]. The typical experiment was carried out by adding the dialyzed ChiNPs to an equal volume of a 1.5 mg/ml solution of hyaluronic acid in 0.1 M acetic buffer (pH 5) with magnetic stirring at 750 rpm for 30 min at room temperature. ChiNPs_PCL were synthesized according to the following protocol [13]. The prepared ChiNPs were concentrated by centrifugation at 16,000 ×*g* for 75 min at 4°C on a 100 μl glycerol bed and dialyzed (Serva, MWCO 3,500 Da).

### Preparation of FITC-Labelled Chitosan

FITC-labelled ChiNPs were prepared using FITC-labelled chitosan as previously described [14]. After preparation, FITC-labelled chitosan was dialyzed using dialysis bags (Serva, MWCO 12,400 Da) against water for 3 days, freeze dried, and stored at -20°C until further use.

### Preparation of Antigen-Loaded Nanoparticles

The loading of Ova to ChiNPs, ChiNPs_HA, and ChiNPs_PCL was carried out by physical adsorption. Briefly, nanoparticles were incubated with Ova in phosphate buffer (0.1 M; pH 7.3) for 2 h. The unbound Ova was collected in the supernatant by centrifugation (1,677 ×*g*) and measured by a micro-BCA assay. To achieve encapsulation of gB antigen within ChiNPs, 150 μg of gB was incorporated into the TPP solution. Tripolyphosphate with gB was slowly added to the chitosan solution under magnetic stirring for 35 min at 600 rpm. The ChiNPs were then separated by centrifugation (1,677 ×*g*) and the protein concentration in the supernatant was measured by a micro-BCA protein assay.

### NP Characterization and Determination of Loading Efficiency and Release Kinetics

The average size of the nanoparticles, the polydispersity index (PI), and the zeta potential in different media were determined by dynamic light scattering (DLS) on a Horiba NanoPartica SZ-100 Series instrument (Horiba, France). Measurements were carried out at 25°C with a scattering angle of 173°. The suspensions of the particles were diluted to a concentration of 0.4 mg/ml in deionized water. All solutions were placed into disposable cells with two openings (Thermo Fisher Scientific, UK). Each measurement was performed 12 times and the results are presented as mean ± SD. ChiNP samples were frozen at -80°C and lyophilized at -110°C for 24 h on an L4-110 Pro (Gregor Instruments, Czech Republic). The Fourier transform infrared (FTIR) spectra of the lyophilized samples were recorded on an FTIR Nicolet iS50 using the ATR technique. A scanning electron microscope (SEM, Tescan, Czech Republic) was used to investigate the microstructure of the sample. The verification of gB loading on nanoparticles was performed by western blot analysis. In brief, anti-M55/gB (MCMV) diluted 1:1000 (Center for Proteomics, Croatia) in blocking buffer (5% non-fat dry milk) was used as primary antibody. Chemiluminiscent detection was performed using SuperSignal West Femto substrate (Thermo Scientific).

The amount of protein adsorbed onto the ChiNPs was calculated by subtracting the protein remaining in the solution after adsorption from the original amount of protein in the reaction using a Micro BCA Protein Assay Kit (Pierce, USA). Entrapment efficiency (EE) and loading content (LC) were calculated using the following equations:

$$\begin{array}{l} \text{EE(\%) = }\frac{\text{Total protein added-Free protein in supernatants}}{\text{Total protein added}}\times 100,\\ \text{LC(\%) = }\frac{\text{Total protein added-Free protein in supernatants}}{\text{Nanoparticles added}}\times 100. \end{array}$$

### Stability Study

The stability of the prepared ChiNPs stored for 14 days at 4°C was analyzed by determining their size, PI, and zeta potential. Samples were characterized on days 0 and 14.

### Cell Cultures

Murine macrophage J774.1 cells were cultured in Dulbecco’s medium (DMEM, Switzerland) with high glucose, containing 10% fetal calf serum, 2 mM glutamine, 100 U/ml penicillin and 100 μg/ml streptomycin. Cells were incubated at 37°C under 5% CO_2_.

### Animals, Immunization and Challenge

Female Balb/c mice (weight 10–20 g; 6–8 weeks old) were purchased from VELAZ (Czech Republic). Animals were housed under veterinary control and standard conditions (light cycle 12 h/12 h, standard laboratory diet and water ad libitum). To determine the immune response, the mice were divided into 5 groups (*n* = 4). Three groups were administered with ChiNPs loaded with gB alone and in combination with adjuvants Poly(I:C) or CpG ODN. The negative control group received saline solution only, and the positive control group was immunized with only gB. The gB was administered by the nasal route at a concentration of 10 μg/mouse. The animals were immunized at week 0, and boosted with the second and third doses at weeks 3 and 6, respectively. The mice were humanely euthanized at week 9 with isofurane (inhalation excess), in order to minimize or avoid animal suffering (Fig. S1). Serum samples were collected and stored at -80°C for further analysis. The spleens were also removed from the mice and splenocyte isolation was immediately performed for flow cytometric analysis. The project was approved by the Institutional Review Board of the Animal Care Committee of the University of Defence (record number MO 18838/2020-684800 and MO 188473/2021-1457), Faculty of Military Health Sciences, Hradec Kralove, Czech Republic. Animals were treated in accordance with the European Convention for the Protection of Vertebrate Animals and in accordance with the ARRIVE Guidelines [15]. All workers who manipulated animals are holders of a Certificate of Professional Competence to Design Experiments and Experimental Trials under the Animal Welfare.

### Detection of MCMV-Specific Antibodies

An in-house ELISA protocol was established to detect IgA and IgG antibodies as follows: MAXISorp plates (NUNC, UK) were coated with 5 μg/ml of recombinant gB in bicarbonate sodium buffer in a volume of 100 μl/well. After an overnight incubation at 4°C, plates were washed 5 times with 0.05% Tween-20 in PBS containing 1%BSA and blocked for 1 h at 37°C with 200 ml of blocking buffer (3% BSA w/v in PBS). Starting at a dilution of 1:25 (for IgA) or 1:100 (IgG), serum samples were serially diluted 1:10 in the dilution buffer (0.1% BSA w/v in PBS) and added in a volume of 100 μl/well. The plates were incubated at 37°C for 1 h, washed 5 times, and the secondary antibody (goat anti-mouse IgG-HRP, goat anti-mouse IgA-HRP; both Invitrogen) was added at a dilution of 1:2000 in the dilution buffer. After incubation at 37°C for 1 h, plates were washed 5 times and developed using 100 μl of substrate solution TMB (3,3',5,5'-Tetramethylbenzidine, Thermo). The reaction was stopped after 15 min with 50 μl of stop solution (2 M H_2_SO_4_) per well. The absorbance was measured on the Synergy plate reader (Biotek, USA) at 450 nm. Endpoint titers were calculated as the reciprocal of the highest dilution giving an absorbance value equal or higher to the average absorbance value for the no-serum control (background).

### In Vitro Luminescent Cell Viability Assay

A viability assay was performed to evaluate the effect of ChiNPs on cells. J774.1 cells were seeded in a 96-well plate (white) at a density of 1.0 × 10^4^ cells/well. Subsequently, ChiNPs were added in the culture medium to obtain the final concentration range of 0.5 to 5 mg/ml. Wells with/without cells in culture medium alone were also included as controls. The cells were subsequently incubated at 37°C under 5% CO_2_ for 24 h. The CellTiter-Glo 2.0 Assay (Promega, USA) was then performed to measure cell viability by quantitation of the ATP level according to manufacturer´s instructions. The luminescence signal was recorded by using a Synergy H1 Multimode Plate Reader (Biotek). Cell viability was expressed as percentage from nontreated cells.

### In Vitro Nanoparticle Uptake by Mononuclear Cells

The ability of ChiNPs to interact with immune cells was tested using FITC-labelled ChiNPs (Ex/Em 490/525 nm), which were prepared as described above. J774.1 cells were seeded in a 24-well plate (Nunclone, UK) at a density of 2.0 × 10^5^ cells/well and incubated at 37°C under 5% CO_2_ overnight. The uptake assay was initiated by adding freshly prepared, labeled ChiNPs to the culture medium into the wells in the following concentrations: 500-250-125-62.5 mg/ml. The plate was incubated for 3 h under the same culture conditions and the cells were fixed with 4% methanol-free paraformaldehyde at room temperature for 15 min. Subsequently, the cells were washed 3 times with PBS and permeabilized using 0.1% Triton X-100 solution for 15 min. The cells were then washed 3 times in PBS and blocked with 1% BSA solution at room temperature for 20 min. Following that, the cells were stained with Alexa-Fluor 680 Phalloidin conjugate (Ex/Em 633/702 nm) at a final concentration of 0.17 μM, and Hoechst 33342 dye (Ex/Em 350/461 nm) at a final concentration of 2 μg/ml for 30 min at room temperature to protect from light. After labeling, the cells were washed twice with PBS, covered with coverslips, and examined using an automated microscope system (Lionheart FX, Biotek) equipped with Gene5 imaging software (Biotek).

### Flow Cytometric Analysis

Mononuclear cells were isolated from mice spleens by Ficoll-Hypaque density gradient centrifugation and the count and viability of the lymphocytes were determined using a Guava EasyCyte Flow Cytometer and Viacount Kit (both Merck, USA). For this, 2 × 10^6^ cells/ml were placed in RPMI containing 10% FBS, 2 mM glutamine, 100 U/ml penicillin, and 100 μg/ml of streptomycin, and with/without 10 μg/ml of recombinant gB. After incubation for 20 h at 37°C, the cells were used for phenotypic analysis of lymphocytes. Then, the cells were washed three times with PBS and viability staining was performed with FVS780 dye (Becton Dickinson, USA) according to the manufacturer’s protocol. Subsequently, the cells were washed and placed in vials containing PBS supplemented with 2% FBS and 0.09% sodium azide (staining buffer). Cell surface marker staining was then performed on the combination with the following conjugated antibodies: CD3-FITC, CD4-PE, CD8-BV650, and CD69-BV421 (all from Becton Dickinson). After incubation in the dark for 30 min on ice, the cells were washed three times with staining buffer. Subsequently, at least 10.000 CD3^+^ events were analyzed by flow cytometric acquisition using the Guava EasyCyte 16ST Flow Cytometer (Merck). Data were analyzed using InCyte software (Merck).

### Data Analysis

Data were analyzed using GraphPad Prism 9 (version 9.20, GraphPad Software Inc., USA). Normality was tested using the Shapiro–Wilk test. Normally-distributed data were analyzed using one-way ANOVA with a post hoc Student’s *t*-test. Non-normally distributed data were analyzed using the Kruskal-Wallis test with the post hoc Mann–Whitney test. Differences were considered significant when *p*<0.05.

## Results

### Nanoparticle Preparation and Characterization

**Fig. 1:**
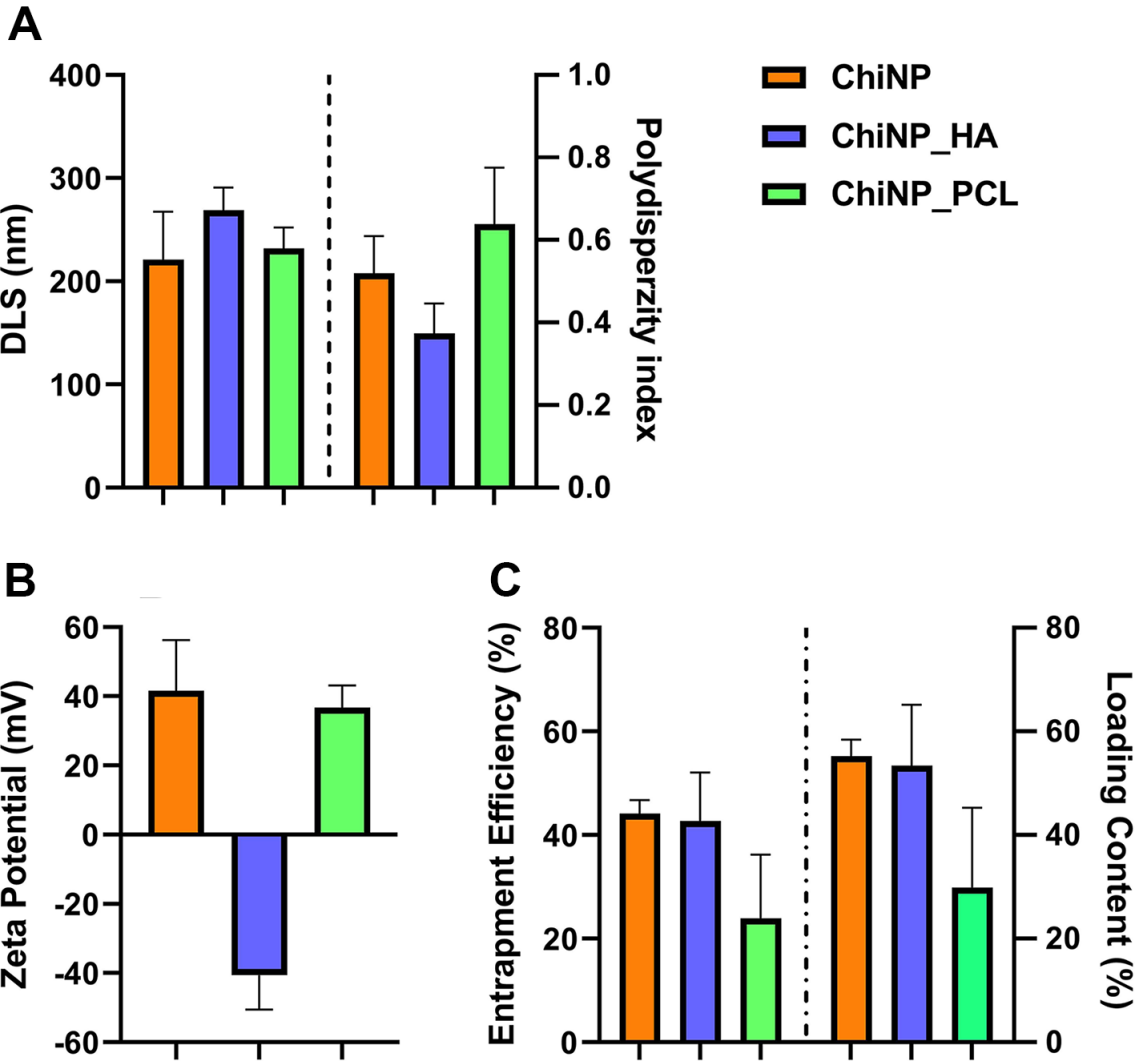
Characterization of synthesized ChiNPs. (**A**) particle size (nm) and PI (**B**) zeta potential (mV), (**C**) entrapment efficiency and loading content. Bars represent the mean ± standard deviation for *n* = 3.

In this study, the chitosan-based nanoparticles were generated by ionotropic gelation of cationic chitosan with TPP. Surface modifications of ChiNPs with hyaluronic acid or PCL were also performed. Characterization of the physicochemical properties showed the following: for ChiNPs, ChiNPs_HA and ChiNPs_PCL, the mean nanoparticle sizes and polydispersity indices were 221.0 ± 46.61 nm (PI: 0.52), 268.8 ± 21.93 nm (PI: 0.37), and 231.9 ± 20.3 (PI: 0.64), respectively (Fig. 1A); for ChiNPs and ChiNPs_PCL the zeta potentials were +41.69 mV and +36.72 mV respectively, while the reversing zeta potential was observed for ChiNPs_HA to a value of -40.64 mV (Fig. 1B). The obtained zeta potential values indicated excellent stability of the nanocarrier in suspension. Subsequently, the ability of ChiNPs to bind the proteins studied was evaluated by an indirect method using the BCA assay. In this study, the Ova and recombinant gB of MCMV were used for the evaluation of the entrapment efficiency (EE) and loading capacity of the ChiNPs. As presented in Fig. 1C, the amounts of protein bound to the ChiNPs were about 44.2%, 42.7%, and 23.9% for ChiNPs, ChiNPs_HA and ChiNPs_PCL respectively. Protein loading content, defined as the ratio of the amount of protein in the nanoparticle to the total amount of NP, was also calculated (Fig. 1C). The verification of gB loading on ChiNPs was evaluated by western blot analysis. The MCMV gB was identified using anti-gB (MCMV) antibody recognizing all molecular mass forms of gB. Supplementary Fig. S2 shows bands corresponding to different molecular masses of gB. The identical bands for the recombinant MCMV gB were detected by Coomassie blue staining (data not shown).

**Fig. 2:**
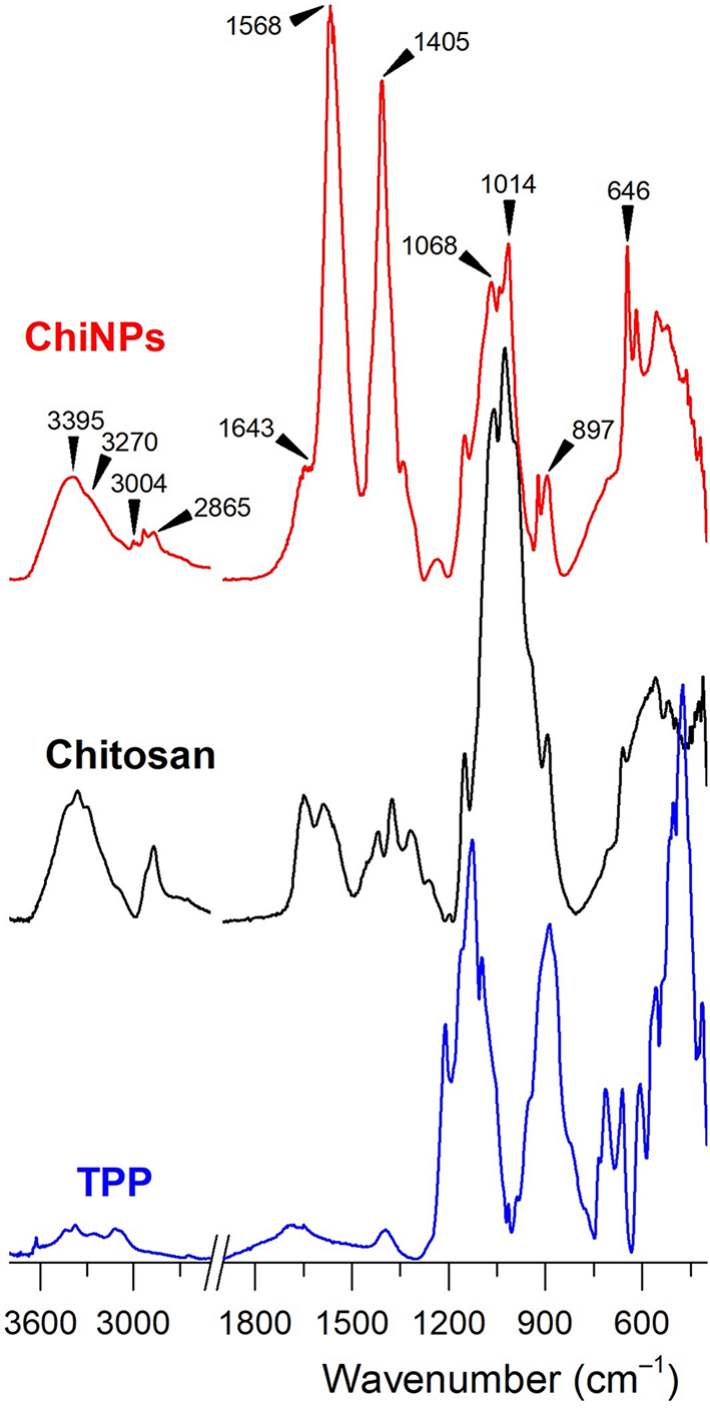
FTIR spectra of ChiNPs, chitosan, and TPP.

Characteristic absorption bands corresponding to various molecular vibrations were recorded in the infrared spectrum of ChiNPs (Fig. 2). The signal at a wavenumber of 3,395 cm^–1^ corresponds to the vibration of the O–H bond, which was typical for the presence of hydroxyl groups in the molecule. The shoulder at frequency 3,270 cm^–1^ was typical for the N–H bond, suggesting the presence of amino groups. In the range of 3,004–2,865 cm^–1^ vibrations of C–H bonds were propagated. The signal at 1,643 cm^–1^ could be interpreted as the amide band originating from the remaining acetamido groups of chitosan (95% degree of deacetylation). The band at frequency 1,568 cm^–1^, combining vibrations of –NH_3_^+^ bending and COO^–^ asymmetric stretching, indicated the presence of protonated amino groups and acetate groups [16]. The COO^–^ symmetric stretching at 1,405 cm^–1^ and COO^–^ bending at 646 cm^–1^ confirmed the presence of acetate anions [16]. The range of 1,200–940 cm^–1^ contained stretching vibrations of C–O bonds as well as P=O. The peak at 897 cm^–1^ corresponded to the vibration of the P–O–P bond stretching, further confirming the presence of the TPP linkage (Fig. 2).

Morphological properties of ChiNPs visualized by SEM are shown in Supplementary Fig. S3. Microscopic analysis verified the nanoparticulate shape and rough surface. The size of individual particles was approximately 200 nm, less than the hydrodynamic size. This can be explained by the loss of their solvation shell [18]. It can be seen that the nanoparticles were rather agglomerated during the analysis.

**Fig. 3:**
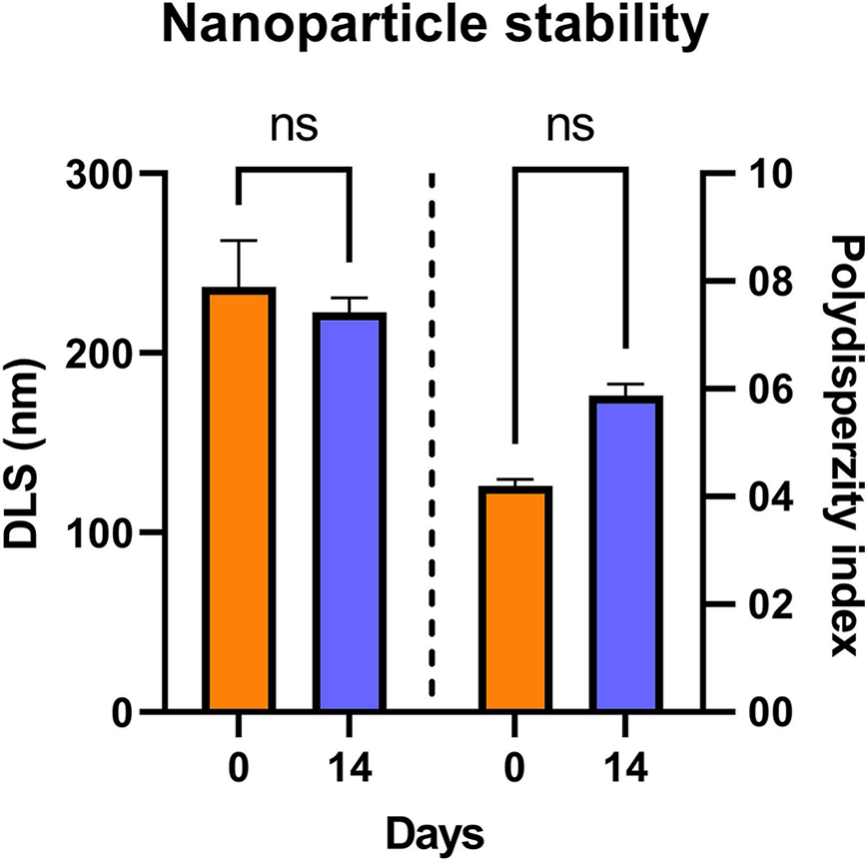
Stability of ChiNPs after 14 days at 4°C. Bars represent the mean ± standard deviation for *n* = 3.

Due to the need for short-term storage prior to analysis and immunization, the prepared ChiNPs were examined for their stability after storage for 14 days at 4°C by measuring their particle size and PI. As illustrated in Fig. 3, there were no significant changes in the particle size, as well as no formation of large aggregates (on the order of micrometers). The increase in the polydispersity index (PI) during storage from 0.4 to 0.6 indicated a decrease in particle stability and broadening of the particle size distribution over time.

### Interaction of ChiNPs with J774.1 Cells

**Fig. 4:**
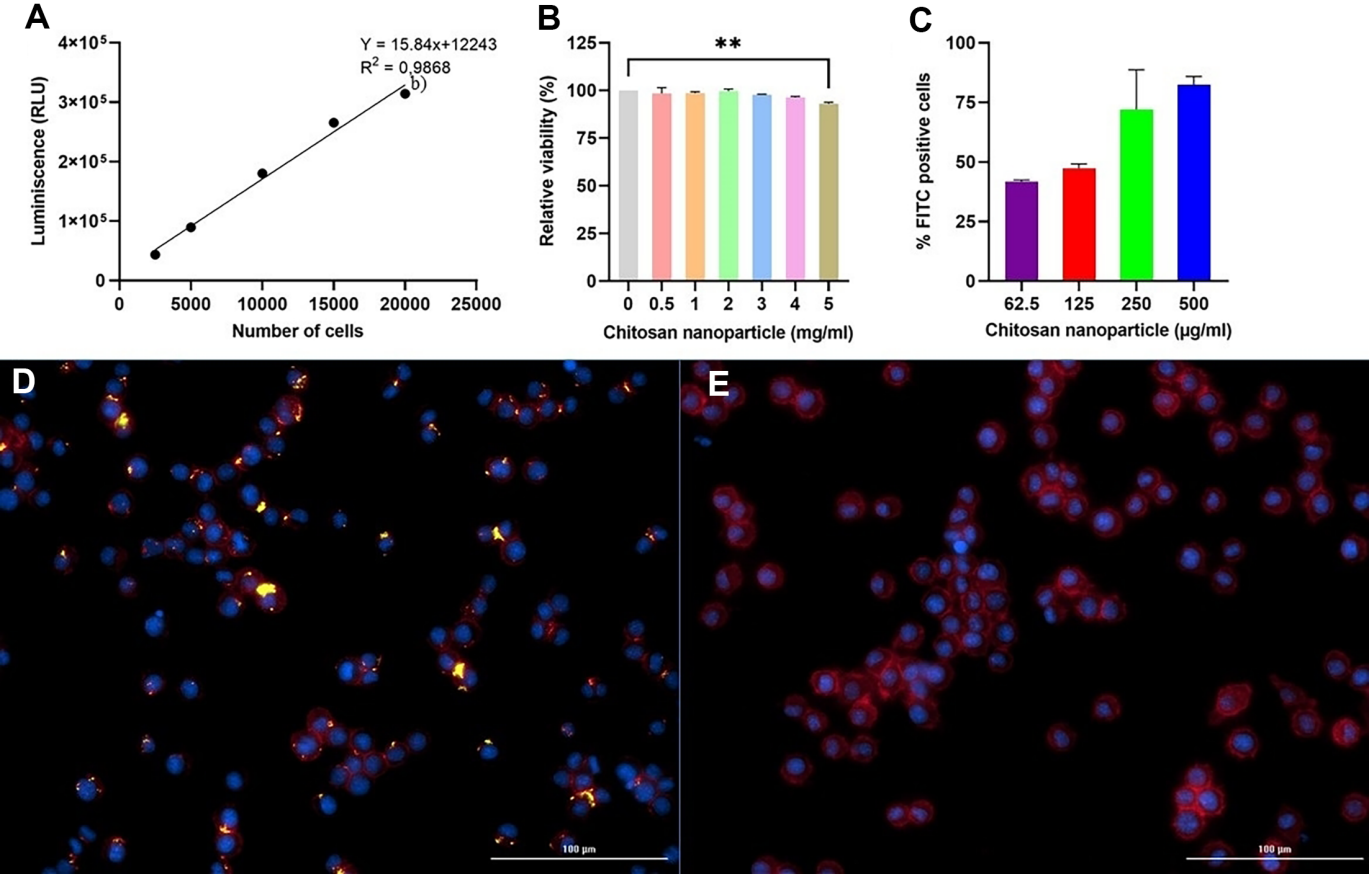
In vitro cell-nanoparticle interaction and cytotoxicity. (**A**), (**B**) Cell viability assay with graded ChiNPs concentration; (**C**), (**D**), and (**E**) The in vitro cellular uptake of FITC-labelled ChiNPs (green) after 3 h. **C**) Dose-dependent efficiency of uptake using increasing concentrations of ChiNPs. **D**) The green dots indicate positive cell uptake. **E**) negative control without labeled ChiNPs. Cell membranes were labeled with Phalloidin-conjugated Alexa Fluor 680 (red) and nuclei with Hoechst 33342 (blue), scale bar = 100 μm, magnification 200X. Chart e) shows the data as mean ± standard deviation.

The cell viability of J774.1 cells was determined in vitro in the presence of ChiNPs. There was no significant drop in the viability of cells tested with the ChiNPs up to a concentration of 4 mg/ml (Fig. 4A and 4B). At higher concentrations of ChiNPs, decreasing viability (*p*<0.01) was observed. Subsequently the effective cellular uptake of ChiNPs was studied by incubating J774.1 cells with various concentrations of nanoparticles for 3 h. The in vitro characteristics of the interaction between ChiNPs and phagocytic cells is presented in Fig. 4. The green fluorescence in Fig. 4D shows that positively stained ChiNPs are phagocytized by the J774.1 cells, compared to the negative control without labeled ChiNPs (Fig. 4E). These results revealed that chitosan-based nanoparticles are readily taken up by phagocytic cells and this uptake is dose dependent up to a concentration of 500 μg/ml (Fig. 4C).

### Immune Response Following Nasal Administration of ChiNP-Based MCMV Vaccine

**Fig. 5:**
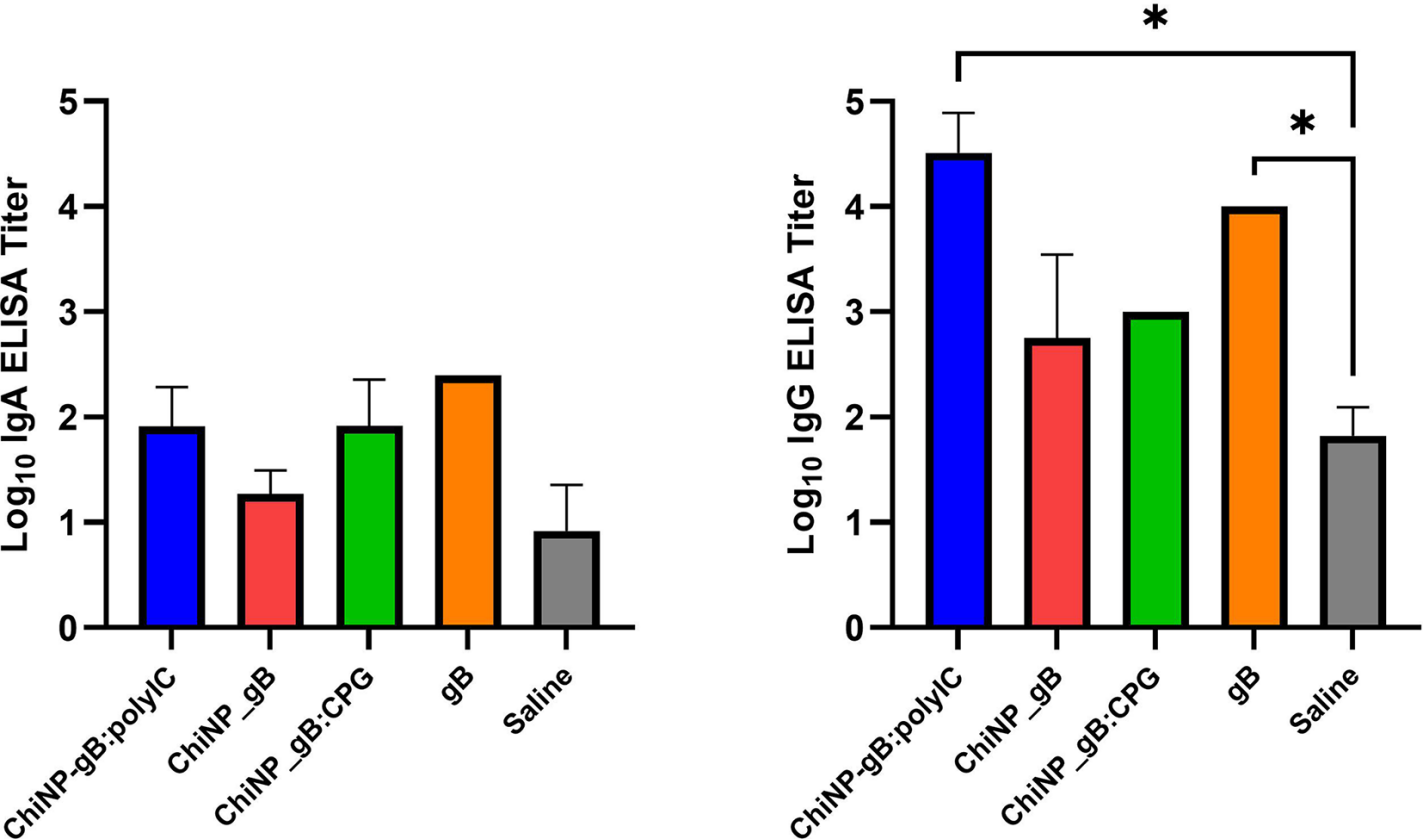
Specific IgA and IgG antibody responses in sera of Balb/c mice after nasal immunization with ChiNPformulated nanovaccine against MCMV. Adjuvants used were CpG ODN, Poly (I:C). Results are presented as mean with SD. Asterisks indicate significant differences between the different adjuvant/antigen groups and Saline control group.

To investigate the ability of ChiNPs to stimulate an antigen-specific immune response, recombinant gB of MCMV was encapsulated into particles. This formulated a nanovaccine, which was then administered to Balb/c mice intranasally. Immunization was performed by the three-dose protocol described in the Materials and Methods section. The antigen-specific humoral and cellular immune responses were subsequently determined. The antigen-specific IgA and IgG endpoint titers from mice serum are shown in Fig. 5. Poly(I:C) and CpG ODN adjuvants have previously been shown to boost induction and generation of antibody levels, and were used in this experiment [19-21].

To evaluate the levels of antigen-specific IgA and IgG in the serum of mice after immunization with ChiNP nanovaccine, we developed and optimized an ELISA (see Materials and Methods). The data presented in Fig. 5 show that levels of vaccine-specific endpoint IgG titers were significantly increased in groups immunized with ChiNP_gB:Poly(I:C) (mean 4.5 logs; *p*<0.05) and gB alone (mean 4 logs; *p*<0.05), compared to the negative control (saline group with mean ELISA titer 1.8 logs). In the ChiNP_gB:CpG ODN and ChiNP_gB groups, the endpoint titer was raised to approximately 1.2 logs above the negative control. The virus-specific IgA antibodies in the serum samples were also quantified using an ELISA. Analyses of sera showed that all of the gB-immunized groups resulted in higher IgA titers compared to the negative control (mean 0.9 log). The use of ChiNP_gB (mean 1.2 logs) immunization elicited only 0.3 log increase in the endpoint titer. The combination with Poly(I:C) or CpG ODN adjuvant (mean 1.9 logs for both) resulted in approximately 1 log increase in the IgA titers. The intranasally administered gB induced the highest increase of IgA titer with mean 2.3 logs.

**Fig. 6:**
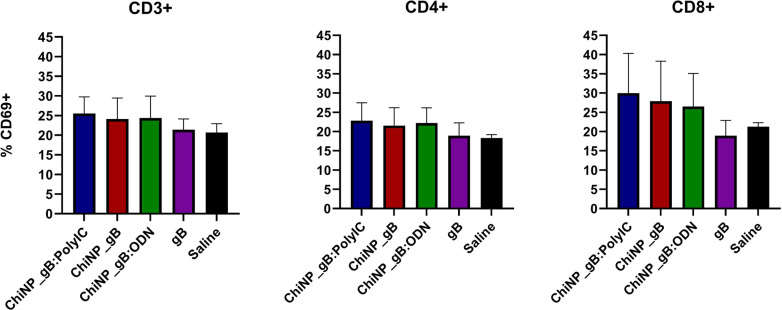
Evaluation of CD69 expression in response to antigen specific in vitro stimulation. Values represent percentages of CD69-positive CD3^+^, CD4^+^, and CD8^+^ detected by flow cytometry.

Subsequently, cell-mediated immune responses leading to cell activation were measured by flow cytometry as the expression of the CD69 activation marker on T cells from immunized mice. The numbers of CD69-positive CD3^+^, CD4^+^, and CD8^+^ cell populations are illustrated in Fig. 6 for each immunized group of animals. The results show that the number of CD3^+^CD69^+^ cells increased slightly (mean range 24-25%) in mice immunized with gB with ChiNPs compared to the gB alone (21%) or negative control (20%). Similar results were obtained for the CD4^+^CD69^+^ and CD8^+^CD69^+^ subpopulations in ChiNP_gB-immunized mice in mean range of 21–22% and 26–29%, respectively. In the control groups immunized with gB alone or with saline solution, these values ranged from 18–21%.

## Discussion

The development of novel effective vaccines is often complicated by instability or low immunogenicity of used antigens that could lead to vaccine failure. This problem mostly occurs in vaccines that are based on peptides or proteins. A promising approach in designing vaccines in the future is to use antigens bound on nanoparticles, which could improve vaccine properties while also inducing additional immune interactions between antigen and immune cells on the mucosal surfaces. Particles derived from natural biopolymers are particularly suitable for biological and clinical applications because of their low toxicity, biocompatibility, and biodegradability [1, 3, 14, 22].

In the present study, ChiNPs were used as a platform for an antigen delivery system. Chitosan as a natural substance has all of the properties listed above. The use of chitosan in vaccines also has another important advantage in that chitosan is well known for its ability to provide positive stimuli for the induction of immune responses [1, 22, 23]. The ChiNPs used in this work had an average size above 200 nm [4, 24]. This diameter was predicted to be applicable because it leads to an efficient uptake by antigen-presenting cells [6, 25]. Measurements of the zeta potential (Fig. 2) showed that ChiNPs and ChiNP_PCL have a positive zeta potential, since solubilization in acidic solutions presents a protonated amino group [4, 14, 24]. When hyaluronic acid is added to ChiNPs, there is an inversion in the zeta potential, because hyaluronic acid occupies most of the positive charges of chitosan due to cross-linking [12, 26]. The positive zeta potential values of ChiNPs and ChiNP_PCL are preferable compared to the negative zeta potential of ChiNP_HA. In addition, better mucoadhesive properties were observed for positively charged nanoparticles. This is due to the presence of a negative charge in mucus and cells [27]. The increased size and lower PI of ChiNP_HA are in accordance with previously described data [26]. According to the physicochemical properties of the tested ChiNPs, the particles derived from chitosan/TPP interaction were considered optimal for further investigation in this study. The results of the FTIR analysis of ChiNPs showed that the spectra were comparable to those of chitosan and TPP. The recorded spectrum of ChiNPs is consistent with those previously published [28].

It is known that instability during the storage or handling of vaccines can lead to physicochemical changes in their formulation and to their degradation, which could negatively affect the efficiency of vaccination. Thus, the short-term stability of prepared ChiNPs using low-molecular-weight chitosan was tested in this study. Similarly to previous reports, slight or no changes in particle size were observed after 2 weeks of storage at 4°C [24]. Analyses such as PI and zeta potential help to evaluate the stability of the nanoparticles. A PI value less than 0.3 gives monodispersity, while a zeta potential of ± 30 mV indicates stability of the nanocarrier in suspension for a longer time. PI represents the distribution of the particle size within a sample. Similar results of PI values (0.4–0.5) for chitosan/TPP particles have been obtained previously [29]. Negatively affected PI values with preserved particle size after storage (from 0.4 to 0.6) indicated a broadening of the polydispersity index. The polydispersity of the particles can limit the efficiency of ligand delivery mediated by these particles. In the polydisperse system, larger particles usually have a higher drug-loading capacity, while smaller particles are expected to have higher efficiency in the delivery of ligands to tissues or cells [30]. On the basis of these results, we decided that the particles for our analyses and immunizations were to be prepared fresh. Very low cytotoxicity of the prepared ChiNPs was also determined using J774.1 cells. These properties support the use of ChiNPs as a delivery system for future application.

Further analyses were conducted to estimate the ability of antigen-presenting cells to effectively uptake ChiNPs, which is critical for inducing potent immune responses. As a model cell line, macrophage-derived J774.1 cells were used. These phagocytic cells are specialized antigen-presenting cells and play an essential role in the induction of both natural and acquired immunity. Therefore, macrophages can also be considered as a potential target for the vaccine. Data in this study show that at least 42% of cells were positive for intracellular localization of ChiNPs after 3 h of incubation. This intracellular delivery is an attractive attribute, especially for the delivery of protein-based vaccines, as it can lead to more robust immune responses involving cytotoxic T cells. This has already been reported for alternative nanocarriers studied for therapeutic vaccine applications [31].

The ability to induce both humoral and cellular immune responses was tested using prepared ChiNPs loaded with gB of MCMV in a mouse model. The immunogenicity of the gB antigen used was demonstrated by the induction of both IgA and IgG antibody titers in immunized animals. The most potent IgG antibody response was observed for the combination of nanoparticle-based vaccine with Poly I:C adjuvant after mucosal delivery. However, the administration of ChiNP-based vaccine did not result in any statistically significant increase of IgA antibody titers after nasal administration.

Cellular immune responses were analyzed by CD69 expression on lymphocytes in immunized mice after antigen specific stimulation. CD69 is a member of the C-type lectin superfamily (Leu-23), which is one of the earliest cell surface antigens expressed by immune cells after their activation [32]. This cell marker has been well documented to be expressed on the surface of lymphocytes within a few hours after stimulation. Our data showed (Fig. 6) higher levels of CD69 expression in groups immunized with gB in combination with ChiNPs. This could be considered as an interesting finding because virus-specific CD8^+^ T cell response is effective in reducing CMV in the host. A similar effect was previously described for ChiNPs in combination with HBsAg after oral vaccination [33].

## Conclusion

A chitosan cationic nanoparticle system carrying a model antigen was established. Although this system showed promising in vitro effects, namely notable antigen uptake by immune cells, this does not reliably predict systemic immune responses in vivo after nasal administration. Future efforts will focus on improving the ability of this system to induce humoral responses and improve cellular immune responses.

## Supplemental Materials

Supplementary data for this paper are available on-line only at http://jmb.or.kr.
