# Supplementary material for: Nasal Immunization Using Chitosan Nanoparticles with Glycoprotein B of Murine Cytomegalovirus
Source: J Microbiol Biotechnol. 2023 Dec 22;34(3):663–72. doi: 10.4014/jmb.2308.08008 (PMC11016792; doi:10.4014/jmb.2308.08008)
Supplement: Supplementary file 1 [file jmb-34-3-663-supple.pdf]

J. Microbiol. Biotechnol.
https://doi.org/10.4014/jmb.2308.08008

Supplementary Figures

Nasal immunization using chitosan nanoparticles with glycoprotein B of murine cytomegalovirus

Day	0	21	42	63	
Prime	x				
Boost 1		x			
Boost 2			x		
Blood				x	ELISA IgA, IgG
Necropsy				x	Flow cytometry

Fig. S1. Time schedule of mice immunization and sample collection.

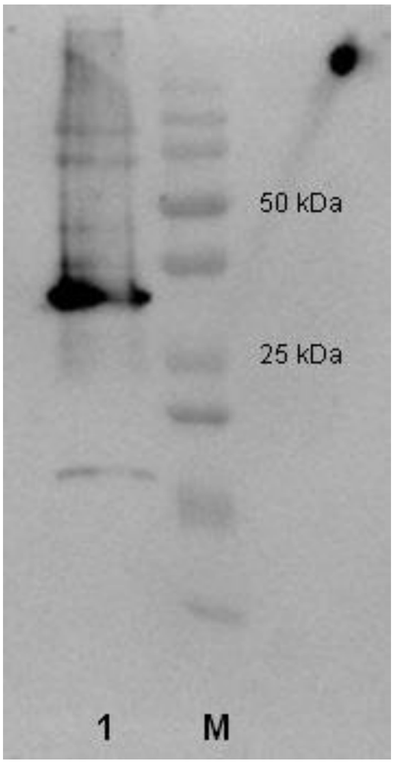

Fig. S2. Western blot analysis of gB-loaded ChiNPs. Lane 1: ChiNPs with loaded gB, Lane M: Protein marker. The MCMV gB was identified using anti-gB (MCMV) antibody recognizing all molecular mass forms of gB.

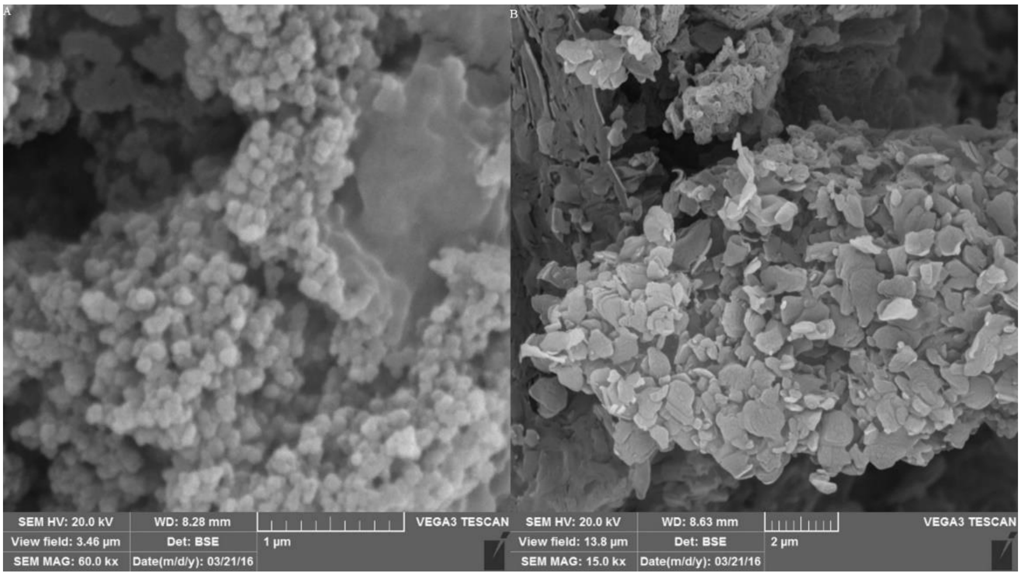

Fig. S3. SEM micrograph analysis of the lyophilised ChiNPs, dialysed in (A) deionised water or (B) acetate buffer pH 5.5. A is reproduced from [17].
